# Functional constraint and small insertions and deletions in the ENCODE regions of the human genome

**DOI:** 10.1186/gb-2007-8-9-r180

**Published:** 2007-09-04

**Authors:** Taane G Clark, Toby Andrew, Gregory M Cooper, Elliott H Margulies, James C Mullikin, David J Balding

**Affiliations:** 1 Department of Epidemiology and Public Health, Imperial College, Norfolk Place, London, W2 1PG, UK; 2 Department of Genetics, Stanford University, Stanford, California 94305, USA; 3 National Human Genome Research Institute, National Institutes of Health, 9000 Rockville Pike, Bethesda, Maryland 20892, USA

## Abstract

**Background:**

We describe the distribution of indels in the 44 Encyclopedia of DNA Elements (ENCODE) regions (about 1% of the human genome) and evaluate the potential contributions of small insertion and deletion polymorphisms (indels) to human genetic variation. We relate indels to known genomic annotation features and measures of evolutionary constraint.

**Results:**

Indel rates are observed to be reduced approximately 20-fold to 60-fold in exonic regions, 5-fold to 10-fold in sequence that exhibits high evolutionary constraint in mammals, and up to 2-fold in some classes of regulatory elements (for instance, formaldehyde assisted isolation of regulatory elements [FAIRE] and hypersensitive sites). In addition, some noncoding transcription and other chromatin mediated regulatory sites also have reduced indel rates. Overall indel rates for these data are estimated to be smaller than single nucleotide polymorphism (SNP) rates by a factor of approximately 2, with both rates measured as base pairs per 100 kilobases to facilitate comparison.

**Conclusion:**

Indel rates exhibit a broadly similar distribution across genomic features compared with SNP density rates, with a reduction in rates in coding transcription and evolutionarily constrained sequence. However, unlike indels, SNP rates do not appear to be reduced in some noncoding functional sequences, such as pseudo-exons, and FAIRE and hypersensitive sites. We conclude that indel rates are greatly reduced in transcribed and evolutionarily constrained DNA, and discuss why indel (but not SNP) rates appear to be constrained at some regulatory sites.

## Abstract 2

Indel rates were observed to be reduced approximately twenty-fold in exonic ENCODE regions, five-fold in sequence that exhibits high evolutionary constraint in mammals and up to two-fold in some classes of regulatory elements.

## Background

Insertion-deletion polymorphisms (indels) have to date received less attention in the study of sequence variation than have single nucleotide polymorphisms (SNPs), despite their frequency (estimated at approximately 16% to 25% of all sequence polymorphism events) and their potential functional importance [1]. 5' Untranslated regions (UTRs) and gene coding regions have previously been observed to have lower indel rates compared with other regions, suggesting that the constraint may have arisen because of negative selection [2]. In general, indels that give rise to frame shifts in coding sequence are more disruptive than non frame-shifts and single point mutations, because of third base degeneracy [3]. As a result, coding sequence indels tend to have lengths that are multiples of three, whereas regulatory sequences tend to have more frequent indels that occur in distinct blocks [4]. The majority of indels are di-allelic and small, with allele length differences of relatively few (one to four) nucleotides [2,5,6]. Given their frequency, small indels could play an important role in contributing to phenotypic differences in humans, including susceptibility to diseases. It is therefore of interest to characterize indel distribution across the human genome, and to integrate indels into SNP marker maps in order to aid in the identification of natural genetic variation.

Recent theoretical work has considered the distribution of indels under neutrality and exploited the evolutionary imprint of sequence indels in order to pinpoint functional DNA regions that are subject to purifying selection [7]. Snir and Pachter [8] used Encyclopedia of DNA Elements (ENCODE) data and multiple primate sequences to study indel events between species. This work suggests that indel rates genome wide are not uniform and that indel events are not neutral; in particular, the work has identified indel hotspots in the human genome. A minority of insertions and deletions may also have plausibly played a major role in speciation events, including human-chimpanzee phenotypic differences [9,10]. An investigation of 2,000 human di-allelic indels found that the majority were monomorphic in chimpanzees and gorillas, indicating that most indels have arisen after the most recent common primate ancestor [6] and are lineage specific [5].

We used the small insertion and deletion ENCODE data [11] to address four questions. First, do the 14 manually selected regions have lower insertion and deletion rates compared with the 30 randomly selected regions? This might be expected to be the case if the selection process [12] for the manually selected ENCODE regions of interest were biased toward regions with greater density of genes or genes of evolutionary importance, with greater functional and evolutionary constraints. Second, do indel rates vary by genomic annotation feature (in turn reflecting varying levels of functional constraint)? Indels that arise in coding sequence are more likely to be deleterious and therefore subject to purifying selection. As a result, DNA sequences that encode proteins might be expected to have some of the lowest genomic indel rates, followed by a wide variety of functional features that are believed to regulate gene expression via an increasing number of previously unrecognized mechanisms [13-17].

Third, are indel rates negatively correlated with measures of evolutionary constraint? We expect indel rates to be negatively associated with evolutionary constraint scores (see Materials and methods, below) where DNA sequences are subject to purifying selection. To address this question, we also correlated indel rates with ancestral repeat (AR) sequence. AR sequences are mobile elements that inserted before the common ancestor of most mammals and have subsequently become inactive [18]. ARs are considered to be predominantly neutral sequences (not subject to purifying selection) and hence we would anticipate indels to accumulate in AR sequence regions with relatively little or no constraint. Based on the assumption that new indels have arisen in AR regions in the past at the same rate as elsewhere in the genome, observed indel rates might be expected to be positively correlated with AR sequence rates.

The fourth question we consider is how do ENCODE indel rates compare with SNP rates across genomic features and evolutionary constrained sequence?

Here we describe the distribution of small indels (ranging from 1 to 20 base pairs [bp]) in the manually and randomly selected ENCODE regions, their distribution in relation to genomic annotation features, and their relationship with measures of evolutionary constraint.

## Results

All identified small indels (*n *= 4486) in the ENCODE regions were mapped onto physical coordinates for ENCODE functional features. The average indel length of identified small indels is 2.8 bp, ranging from 1 to 20 bp. The overall density is on average 15 indels per 100 kilobases (kb; 99% confidence interval [CI] 13.4 to 16.7) or, in terms of total indel length, 43.4 bp per 100 kb (99% CI 38.3 to 49.1). All results in Tables 1 to 3 are presented in two ways: as numbers of indel events (indels per 100 kb) and total indel length (indel bp per 100 kb). In the interests of brevity, indel rates are referred to in the text to as indel bp per 100 kb unless stated otherwise. This also facilitates comparison with SNP rates.

**Table 1:** Indel density (for all 44 ENCODE regions)

ENCODE region	Chromosome	Number of indels	Indels (bp)	Size (bp)	Density (per 100 kb)	Density (bp per 100 kb)	Gene (bp%)	Val. SNP (per 100 kb)	SNP:indel	
									
									(per 100 kb)	(bp/100 kb)
**Overall**		**4,486**	**13,010**	**29,998,060**	**15.0**	**43.4**	**2.2**	**102.4**	**6.7**	**2.4**
										
1: ENm001 CFTR	7	189	533	1,877,426	10.1	28.4	1.2	64.5	5.8	2.3
2: ENm002 Interleukin	5	139	535	1,000,000	13.9	53.5	3.0	101.1	6.6	1.9
3: ENm003 ApoCluster	11	59	187	500,000	11.8	37.4	2.1	93.2	8.4	2.5
4: ENm004	22	289	789	1,700,000	17.0	46.4	2.1	89.7	4.9	1.9
5: ENm005	21	368	982	1,695,985	21.7	57.9	2.4	108.1	4.3	1.9
6: ENm006	X	97	249	1,338,447	7.2	18.6	5.5	34.5	7.4	1.9
7: ENm007	19	207	711	1,000,876	20.7	71.0	4.9	151.6	7.8	2.1
8: ENm008 AlphaGlobin	16	118	253	500,000	23.6	50.6	5.2	120.2	5.0	2.4
9: ENm009 BetaGlobin	11	168	545	1,001,592	16.8	54.4	4.2	181.4	10.9	3.3
10: ENm010 HOXACluster	7	95	317	500,000	19.0	63.4	2.4	89.4	4.5	1.4
11: ENm011 1GF2H19	11	62	228	606,048	10.2	37.6	2.1	102.3	13.4	2.7
12: ENm012 FOXP2	7	128	370	1,000,000	12.8	37.0	0.3	73.2	5.9	2.0
13: ENm013	7	139	483	1,114,424	12.5	43.3	1.0	105.7	7.7	2.4
14: ENm014	7	128	322	1,163,197	11.0	27.7	0.8	83.4	7.6	3.0
										
**Manual**		**2,186**	**6,504**	**14,997,995**	**14.6**	**43.4**	**2.7**	**95.9**	**6.5**	**2.2**
										
15: ENr111	13	96	364	500,000	19.2	72.8	0.3	128.2	5.7	1.8
16: ENr112	2	55	156	500,000	11.0	31.2	0.0	94.2	10.6	3.0
17: ENr113	4	56	152	500,000	11.2	30.4	0.1	104.0	9.6	3.4
18: ENr114	10	101	284	500,000	20.2	56.8	1.0	142.8	8.5	2.5
19: ENr121	2	108	270	500,000	21.6	54.0	0.8	140.0	6.1	2.6
20: ENr122	18	76	287	500,000	15.2	57.4	1.9	139.8	8.2	2.4
21: ENr123	12	65	136	500,000	13.0	27.2	2.5	122.0	9.2	4.5
22: ENr131	2	75	202	500,064	15.0	40.4	3.6	123.4	6.9	3.1
23: ENr132	13	43	169	500,000	8.6	33.8	1.9	123.8	14.5	3.7
24: ENr133	21	112	293	500,000	22.4	58.6	2.2	165.0	6.3	2.8
25: ENr211	16	68	251	500,001	13.6	50.2	0.1	114.8	8.8	2.3
26: ENr212	5	70	118	500,000	14.0	23.6	0.3	112.6	7.6	4.8
27: ENr213	18	74	165	500,000	14.8	33.0	0.6	91.8	6.0	2.8
28: ENr221	5	68	156	500,000	13.6	31.2	1.4	105.0	6.9	3.4
29: ENr222	6	73	201	500,000	14.6	40.2	0.9	104.0	6.4	2.6
30: ENr223	6	130	384	500,000	26.0	76.8	2.2	135.2	4.5	1.8
31: ENr231	1	91	178	500,000	18.2	35.6	4.8	94.6	4.9	2.7
32: ENr232	9	93	282	500,000	18.6	56.4	3.2	112.0	5.8	2.0
33: ENr233	15	47	126	500,000	9.4	25.2	7.3	59.8	7.4	2.4
34: ENr311	14	50	171	500,000	10.0	34.2	0.0	93.0	8.8	2.7
35: ENr312	11	54	176	500,000	10.8	35.2	0.0	142.0	12.2	4.0
36: ENr313	16	83	242	500,000	16.6	48.4	0.0	108.4	6.4	2.2
37: ENr321	8	84	257	500,000	16.8	51.4	0.4	94.0	4.6	1.8
38: ENr322	14	86	323	500,000	17.2	64.6	0.8	127.6	7.3	2.0
39: ENr323	6	77	176	500,000	15.4	35.2	0.7	78.8	4.7	2.2
40: ENr324	X	70	138	500,000	14.0	27.6	1.3	43.8	4.3	1.6
41: ENr331	2	67	204.0	500,000	13.4	40.8	6.4	118.8	8.4	2.9
42: ENr332	11	60	184	500,000	12.0	36.8	6.5	88.6	7.6	2.4
43: ENr333	20	89	226	500,000	17.8	45.2	6.1	77.2	4.0	1.7
44: ENr334	6	79	235	500,000	15.8	47.0	2.2	83.4	5.4	1.8

**Random**		**2,300**	**6,506**	**15,000,065**	**15.3**	**43.4**	**2.0**	**109.0**	**6.9**	**2.5**

Manual (regions 1-14; each approx. 500 kb-2 MB) and random (regions 15-44 each approx. 500 kb) selected ENCODE regions are defined [12] as:

Manual: genomic regions with well studied genes and availability of comparative sequence

Random: selected randomly across the genome, stratified by gene density and non-exonic conservation

The ten Encyclopedia of DNA Elements (ENCODE) regions with in-depth single nucleotide polymorphism (SNP) discovery are ENm010, ENm013, ENm014, ENr112, ENr113, ENr123, ENr131, ENr213, ENr232, and ENr321. bp, base pairs; kb, kilobases.

**Table 2:** Indel density for annotation features (across all 44 ENCODE regions)

	Indels		Rate (number per 100 kb)		Rate (bp per 100 kb)		
		
	*n*	bp	*n*	99% CI	bp	99% CI	Feature length (kb)
Manual	2,186	6,504	14.6	11.7 to 18.2	43.4	34.4 to 54.7	14,998
Random	2,300	6,506	15.3	13.6 to 17.3	43.4	37.5 to 50.2	15,000
Overall	4,486	13,010	15.0	13.4 to 16.7	43.4	38.3 to 49.1	29,998
							
RNA transcription							
CDS	5	5	0.7	0.1 to 8.6	0.7	0.1 to 8.6	675
TSS	2	2	3.3		3.3		61
RACEfrags	9	28	2.1	0.8 to 5.4	6.6	1.3 to 33.9	425
TARs/transfrags	37	78	5.8	3.5 to 9.6	12.3	6.8 to 22.3	634
Pseudo-exons	9	26	6.6	2.6 to 16.6	19.1	5.8 to 63.3	136
3' UTR	48	103	11.0	7.2 to 16.7	23.6	13.5 to 41.3	436
5' UTR	7	32	6.0	1.6 to 22.3	27.4	3.8 to 198.7	117
TUF	53	160	12.2	7.8 to 19.2	36.9	20.2 to 67.6	433
							
Open chromatin							
FAIRE-sites	106	327	7.7	5.6 to 10.6	23.8	15.5 to 36.7	1,372
DHS (NHGRI)	19	61	6.1	3.3 to 11.3	19.7	8.3 to 46.9	310
DHS (Regulome)	43	135	8.6	5.3 to 14.0	27.0	13.4 to 54.4	499
							
DNA-protein intreraction/transcript regulation							
HisPolTAF	141	348	13.1	10.0 to 17.2	32.4	22.5 to 46.5	1,076
Seq_specific (all motifs)	131	420	11.2	8.3 to 15.0	35.8	23.1 to 55.3	1,174
SeqSp (sequence specific factors)	54	225	10.2	6.2 to 16.7	42.5	20.1 to 89.5	530
							
Ancestral repeats	532	1,592	7.9	6.7 to 9.2	26.5	21.7 to 32.5	5,998
							
Evolutionary constraint							
MCS strict	19	31	2.5	1.3 to 5.1	4.1	1.6 to 10.4	748
MCS moderate	78	170	5.1	3.5 to 7.6	11.2	6.8 to 18.5	1,515
MCS loose	356	960	9.8	8.2 to 11.7	26.4	20.9 to 33.4	3,637
							
Cell cycle							
EarlyRepSeg	1,124	2,989	16.4	13.8 to 19.4	43.5	33.3 to 56.9	6,868
MidRepSeg	1,190	3,352	15.4	13.5 to 17.5	43.2	35.3 to 53.0	7,751
LateRepSeg	1,110	3,345	13.9	12.1 to 15.9	41.9	32.9 to 53.3	7,991

bp, base pairs; CDS, coding sequence; CI, confidence interval; DHS, DNAse hypersensitive sites; ENCODE, Encyclopedia of DNA Elements; FAIRE, formaldehyde assisted isolation of regulatory elements; kb, kilobases; MCS, multi-species conserved sequence; NHGRI, National Human Genome Research Institute; transfrag, transcribed fragment; RACEfrag, rapid amplification of cDNA ends fragment; TAR, transcriptionally active region; TSS, transcription start site; TUF, transcripts of unknown function; UTR, untranslated region.

**Table 3:** Comparison of indel and SNP density by ENCODE experimental features

	Indels		Validated SNPs	
	
	bp/100 kb	99% CI	bp	bp/100 kb
Manual	43.4	34.4 to 54.7	14,390	95.9
Random	43.4	37.5 to 50.2	16,343	109.0
Overall	43.4	38.3 to 49.1	30,733	102.4
				
RNA transcription				
CDS	0.7	0.1 to 8.6	421	62.4
TSS	3.3		42	68.7
RACEfrags	6.6	1.3 to 33.9	278	65.4
TARs/transfrags	12.3	6.8 to 22.3	591	93.1
Pseudo-exons	19.1	5.8 to 63.3	132	96.9
3' UTR	23.6	13.5 to 41.3	370	84.8
3' UTR	27.4	3.8 to 198.7	97	83.2
TUF	36.9	20.2 to 67.6	423	97.6
				
Open chromatin				
FAIRE-sites	23.8	15.5 to 36.7	1,232	89.8
DHS (NHGRI)	19.7	8.3 to 46.9	297	95.9
DHS (Regulome)	27.0	13.4 to 54.4	450	90.1
				
DNA-protein interaction/transcript regulation				
HisPolTAF	32.4	22.5 to 46.5	850	79.0
Seq_specific (all motifs)	35.8	23.1 to 55.3	1,098	93.5
SeqSp (sequence specific factors)	42.5	20.1 to 89.5	421	79.4
				
Ancestral repeats	26.5	21.7 to 32.5	5,749	95.9
				
Evolutionary constraint				
MCS strict	4.1	1.6 to 10.4	229	30.6
MCS moderate	11.2	6.8 to 18.5	667	44.0
MCS loose	26.4	20.9 to 33.4	2,052	56.4
				
Cell cycle				
EarlyRepSeg	43.5	33.3 to 56.9	6,165	89.8
MidRepSeg	43.2	35.3 to 53.0	7,418	95.7
LateRepSeg	41.9	32.9 to 53.3	8,896	111.3

bp, base pairs; CDS, coding sequence; CI, confidence interval; DHS, DNAse hypersensitive sites; ENCODE, Encyclopedia of DNA Elements; FAIRE, formaldehyde assisted isolation of regulatory elements; kb, kilobases; MCS, multi-species conserved sequence; NHGRI, National Human Genome Research Institute; transfrag, transcribed fragment; RACEfrag, rapid amplification of cDNA ends fragment; SNP, single nucleotide polymorphism; TAR, transcriptionally active region; TSS, transcription start site; TUF, transcripts of unknown function; UTR, untranslated region.

There are no substantial differences in indel or gene density between manually and randomly selected regions (Table 1). The indel rates in manual regions are similarly variable (sd_num/100 kb _= 5.0 number of indels per 100 kb; sd_bp/100 kb _= 14.7 indel bp per 100 kb, where sd_num/100 kb _and sd_bp/100 kb _refer to the standard deviation for number of indels and indel bp per 100 kb, respectively) to those in random regions (sd_num/100 kb _= 4.0; sd_bp/100 kb _= 14.0), with no significant differences in the summary data (F_[13,29] _= 1.52, *P *= 0.34).****

We observed a reduction in indel rates for coding sequence and annotation features that are believed to play a regulatory role in gene expression (Table 2). Compared with the overall mean (43.4 bp per 100 kb), ENCODE coding sequences all exhibit a significant reduction in indel rates, as assessed by identifying open reading frames (coding sequence [CDS] mean indel rate: 0.7 bp per 100 kb), transcription start sites (TSSs; 3.3 bp per 100 kb), rapid amplification of cDNA ends fragments (RACEfrags; 6.6 bp per 100 kb), and transcribed fragments (12.3 bp per 100 kb). Pseudo-exons (19.1 bp per 100 kb), 3' UTRs (23.6 bp per 100 kb), 5' UTRs (27.4 bp per 100 kb), and transcripts of unknown function (36.9 bp per 100 kb) all exhibit a reduction in indel rates compared with the overall mean for all ENCODE sequence, but these findings are not statistically significant.

Potential regulatory elements, assessed by measuring open chromatin sites, also reveal sequences with constrained indel rates (Table 2). Formaldehyde assisted isolation of regulatory elements (FAIRE) sites (23.8 bp per 100 kb) and DNAse hypersensitive sites (DHS; [NHGRI group] 19.7 bp per 100 kb and [Regulome group] 27.0 bp per 100 kb) both exhibit reduced indel rates. DHS are short regions of DNA that are relatively easily cleaved by DNAse I.

Acetylated histones are usually associated with transcriptionally active chromatin and deacetylated histones with inactive chromatin. Hence, histone modified regions often signify regulatory sites. Selected histone modifications and binding sites for RNA polymerase II and the general transcription factor TAF250 were assayed for the ENCODE regions (see ENCODE Project Consortium [19] and Table 4 for details). These sites show modestly reduced indel rates (HisPolTAF: 32.4 bp per 100 kb), along with sites occupied by sequence specific binding proteins (all motifs: 35.8 bp per 100 kb), but neither finding is statistically significant.

**Table 4:** Experimental feature definitions

Feature	Term	Definition
RNA transcription (coding and noncoding)	CDS	Coding sequence: well characterized transcribed regions with an annotated protein-coding open reading frame (ORF)
	RACEfrags	5' and 3' rapid Amplification of cDNA ends (RACE), using polyA or total RNA to construct full-length cDNA. This technique has revealed previously unrecognized UTRs
	TARs/transfrags	Transcriptionally active regions/transcribed fragments as determined by analyses of cellular RNA (polyA or total) hybridizations to multiple microarray platforms. For the analyses reported here, portions of TARs/transfrags overlapping any CDS, 5' or 3' UTR annotations were removed from the dataset
	Pseudo-exons	A pre-mRNA sequence that resembles an exon but is not recognized as such by the splicing machinery
	TSS	Transcription start site
	5' UTR	Untranslated region: portions of CDS-containing transcripts before the start codon. For the analyses reported here, 5' UTRs overlapping alternatively transcribed CDS annotations were removed from the dataset
	TUF	Transcripts of unknown function for noncoding transcripts
	3' UTR	Untranslated region: portions of CDS-containing transcripts after the stop codon
Transcript regulation: open chromatin/DNA-protein interaction	DHS	DNAse I hypersensitive sites are short regions of DNA that are relatively easily cleaved by deoxyribonuclease. Regions of open chromatin detected by quantitative chromatin profiling and novel microarray-based methods. For the analyses reported here, regions that overlap repetitive sequence were removed. Measures of DHS are reported using two sources: the ENCODE Regulome group and the NHGRI
	FAIRE-sites	Formaldehyde assisted isolation of regulatory elements: a procedure used to isolate chromatin that is resistant to the formation of protein-DNA crosslinks. Data suggest that depletion of nucleosomes (the most basic organizational unit of chromatin) at active regulatory regions, such as promotors, is the primary underlying basis for FAIRE [38]
	HisPolTAF	Histone modifications, RNA polymerase II (PolII), and transcription regulator TAF250
	Sequence specific factors	Regions of DNA determined to be bound by sequence-specific transcription factors through chromatin immunoprecipitation followed by microarray chip hybridization (so-called 'ChIP-Chip') analyses
	Sequence specific (all motifs)	Computationally identified short sequence motifs found to be over-represented in the sequence specific factors dataset
Ancestral repeats		Mobile elements with well defined consensus sequences that inserted into the ancestral genome prior to mammalian radiation. These sequences are considered to be predominantly non-functional and are often used as models of neutrally evolving DNA
Cell cycle	EarlyRepSeg	Early replicating segments
	MidRepSeg	Mid replicating segments
	LateRepSeg	Late replicating segments
Evolutionary constraint	MCS strict	Multi-species conserved sequences: strict criteria
	MCS moderate	Multi-species conserved sequences: modest criteria
	MCS loose	Multi-species conserved sequences: loose criteria

Multi-species constrained sequence (MCS moderate; 11.2 bp per 100 kb) show greatly reduced indel rates (Table 2), similar to rates in coding regions. AR regions (26.5 bp per 100 kb) also showed unexpectedly reduced indel rates. Cell cycle replicating segments (MidRepSeg: 43.2 bp per 100 kb) show no relationship with indel rates.

**Figure 1:**
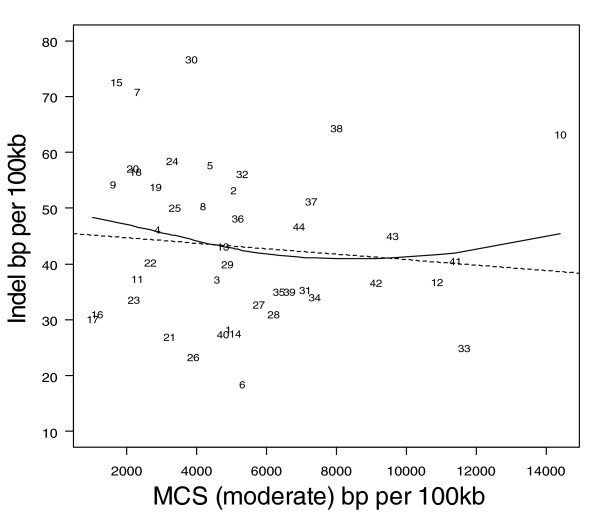
Indel rate versus MCS modest for human and 13 mammals. Indel rate and multi-species constrained sequences (MCS modest) are both expressed as base pairs (bp) per 100 kilobases (kb). The solid line represents the fit from a cubic smoothing spline, whereas the dashed line is the fit from a robust linear regression.

**Figure 2:**
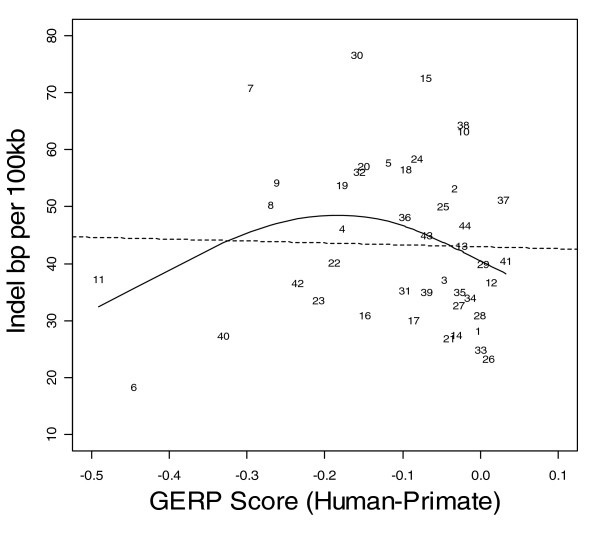
Indel rate versus GERP score comparing human and primates. Indel rate is expressed as base pairs (bp) per 100 kilobases (kb). The solid line represents the fit from a cubic smoothing spline, whereas the dashed line is the fit from a robust linear regression. GERP, genomic evolutionary rate profiling.

**Figure 3:**
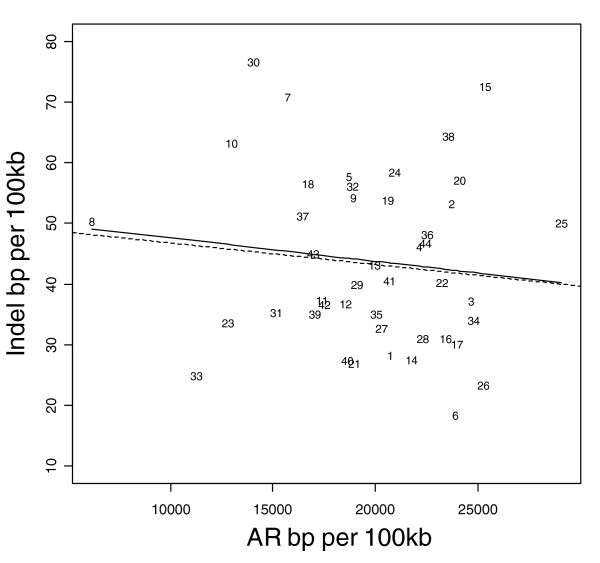
Indel rate versus all AR sequence rate. Indel rate and ancestral repeat (AR) sequence rate are both expressed as base pairs (bp) per 100 kilobases (kb). The solid line represents the fit from a cubic smoothing spline, whereas the dashed line is the fit from a robust linear regression. Note that the same relationship is observed for indel rate versus long AR bp per 100 kb.

Figures 1 to 3 show the relationship between indel base pairs per 100 kb and measures of mammalian evolutionary constraint, human-primate evolutionary constraint, and AR rates, with each data point representing a summary score for each ENCODE region. The Pearson correlation coefficients relating to Figures 1 to 3 are statistically insignificant when all of the ENCODE region summary data points are considered. However, when outlying data points are identified and excluded using standard regression diagnostics, the correlations are of marginal statistical significance. Indel rates are (nonsignificantly) inversely correlated with mammalian MCS score (Figure 1; *r *= -0.25, *P *= 0.11 with outlier ENCODE region 10 excluded), and negatively associated with the primate genomic evolutionary rate profiling (GERP) score and GERP squared using multiple regression (Figure 2; multiple correlation coefficient: *R *= 0.32, *P *= 0.04). Indel rates are also observed to be marginally and negatively correlated with AR rates and AR squared (Figure 3; multiple correlation coefficient: *R *= -0.30, *P *= 0.06 with regions 8 and 15 identified as outliers).

**Figure 4:**
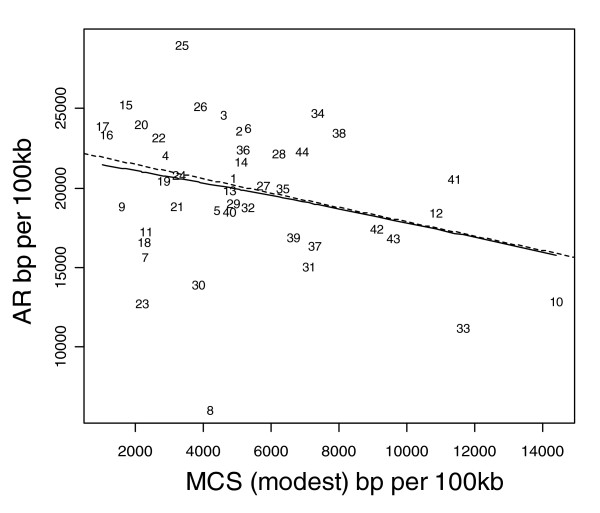
AR sequence rate versus MCS modest. Ancestral repeat (AR) sequence rate and multi-species conserved sequences (MCS modest) are both expressed as base pairs (bp) per 100 kilobases (kb). The solid line represents the fit from a cubic smoothing spline, whereas the dashed line is the fit from a robust linear regression.

**Figure 5:**
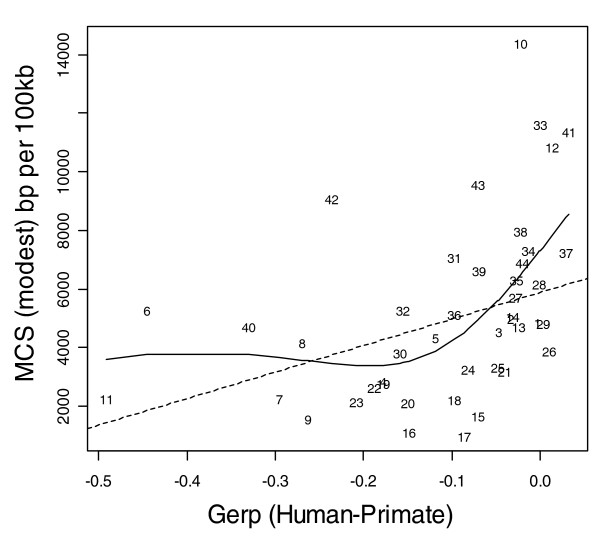
MCS modest versus GERP human-primate score. Multi-species conserved sequences (MCS modest) is expressed as base pairs (bp) per 100 kilobases (kb). The solid line represents the fit from a cubic smoothing spline, whereas the dashed line is the fit from a robust linear regression. GERP, genomic evolutionary rate profiling.

AR rates (bp per 100 kb) are strongly inversely correlated with MCS (Figure 4; *r *= -0.46, *P *< 0.002), but exhibit no relation with either human-primate or human-mammal GERP scores (plots not shown; GERP primate: *r *= 0.02, *P *= 0.91; GERP mammal: *r *= -0.03, *P *= 0.8). MCS and GERP constraint scores are positively correlated with one another in a curvilinear relationship (Figure 5; *r *= 0.42, *P *= 0.005), with the homeobox gene family HOXA cluster, ENCODE region 10, identified as a highly conserved outlier region on the MCS but not an outlier on either of the GERP scores.

**Figure 6:**
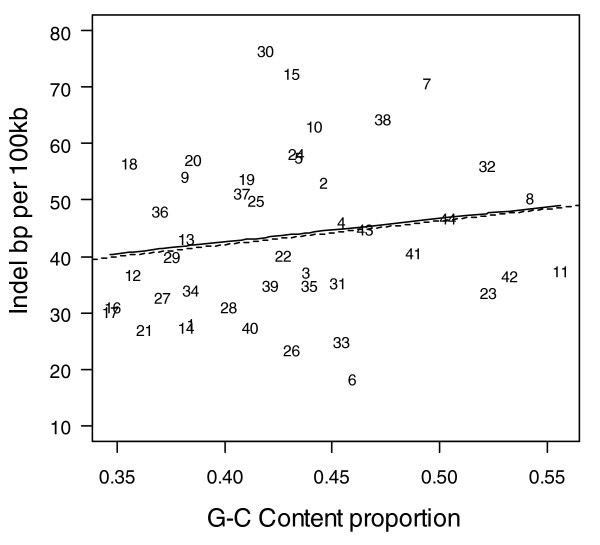
AR sequence rate versus GC content. Ancestral repeat (AR) sequence rate is expressed as base pairs (bp) per 100 kilobases (kb). The reduced local GC content observed in AR sequence reflects the process of deamination of methylated CpG to TpG dinucleotides in vertebrate sequence over long evolutionary periods of time [3]. The solid line represents the fit from a cubic smoothing spline, whereas the dashed line is the fit from a robust linear regression.

**Figure 7:**
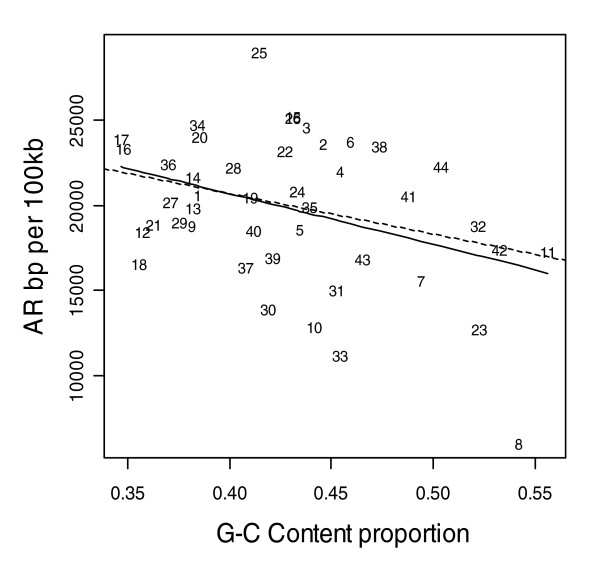
Indel rates versus GC content. Indel rate is expressed as base pairs (bp) per 100 kilobases (kb). The solid line represents the fit from a cubic smoothing spline, whereas the dashed line is the fit from a robust linear regression.

AR rates also exhibit a strong negative correlation with local GC content (Figure 6; *r *= -0.55, *P *= 0.001). Indel rates show an overall positive correlation with GC content for the ENCODE regions (Figure 7), which illustrates that indel rates may be confounded by local GC content. In order to check the effect of GC content on indel rates, we recalculated the results presented in Table 2 including GC content as a confounder. For example, although indel events per 100 kb in AR sequence is observed to be about 7.9 (99% CI 6.7 to 9.2; see Table 2), the mean rates are about 4.7 (99% CI 3.5 to 6.4) and about 10.4 (99% CI 8.6 to 12.4) for AR sequence with GC content above 50% and GC content below 50%, respectively. However, the mean indel rates presented in Table 2 are not significantly altered when adjusted for local GC content at each annotational feature (data not presented).

Table 3 compares the distribution of indel and validated SNP rates by experimental feature. In general, indel rates are lower than SNP rates, with a ratio of validated SNPs to indel event rates of 6.7 (102.4/15), or 2.4 (102.4/43.4) for validated SNPs:indel bp. The pattern of indel rates across genomic features is broadly similar to SNP density. For example, as a percentage of their respective overall means, the indel rates for MCS evolutionary constraints of strict, moderate, and loose are 10%, 26% and 61%, compared with 29%, 43% and 55% for SNP rates. Similarly, the indel and SNP rates are reduced for many transcribed sequences (CDS, TSS, and RACEfrags).

For some features, however, the pattern of constraint for indel and SNP rates differ quite markedly (Table 3). Although indel rates are constrained in chromatin mediated transcription regulatory sites (FAIRE: 23.8 bp per 100 kb; DHS: 19.7 to 27.0 bp per 100 kb), SNP rates are not constrained for these features (FAIRE: 90 SNPs per 100 kb; DHS: 90 to 96 SNPs per 100 kb) as compared with the overall mean (102.4 SNPs per 100 kb).

Table 5 compares indel rates by functional annotation for these data and the data presented by Bhangale and coworkers [20]. The overall indel rates are very similar for indel events (15 per 100 kb versus 13.8 per 100 kb for the data presented by Bhangale and coworkers [20]) and indel bp (43.4 bp per 100 kb versus 39.4 bp per 100 kb). The indel rates presented by Bhangale and coworkers [20] are also greatly reduced for coding DNA but not pseudo-exons or UTR sequence. Open chromatin indel rates are reduced in both datasets.

**Table 5:** Comparison of ENCODE and Bhangale *et al*. (ten ENCODE regions) indel data

	ENCODE (44 ENCODE regions/Baylor)				Bhangale *et al*. (ten ENCODE regions/Baylor)			
	
	Indels		Rate (per 100 kb)		Indels		Rate (per 100 kb)	
	
	*n*	bp	*n*	bp	*n*	bp	*n*	bp
Manual	2,186	6,504	14.6	43.4	362	1,122	13.0	40.4
Random	2,300	6,506	15.3	43.4	502	1,350	14.3	38.6
Overall	4,486	13,010	15.0	43.4	864	2,472	13.8	39.4
								
RNA transcription								
CDS	5	5	0.7	0.7	1	1	1.2	1.2
TSS	2	2	3.3	3.3	0	0	0.0	0.0
RACEfrags	9	28	2.1	6.6	0	0	0.0	0.0
TARs/transfrags	37	78	5.8	12.3	6	11	7.5	13.7
Pseudo-exons	9	26	6.6	19.1	2	10	9.7	48.7
3' UTR	48	103	11.0	23.6	11	29	18.7	49.2
5' UTR	7	32	6.0	27.4	4	8	37.3	74.6
TUF	53	160	12.2	36.9	4	18	8.1	36.4
								
Open chromatin								
FAIRE sites	106	327	7.7	23.8	17	72	5.6	23.6
DHS (NHGRI)	19	61	6.1	19.7	1	1	2.8	2.8
DHS (Regulome)	43	135	8.6	27.0	15	40	8.5	22.6
								
DNA-protein intreraction/transcript Regulation								
HisPolTAF	141	348	13.1	32.4	32	114	12.8	45.5
Seq_specific (all motifs)	131	420	11.2	35.8	28	122	33.4	145.3
SeqSp (sequence specific factors)	54	225	10.2	42.5	9	45	5.1	25.6
								
Ancestral repeats	532	1,592	7.9	26.5	110	280	8.7	22.1
								
Evolutionary constraint								
MCS strict	19	31	2.5	4.1	5	9	3.3	5.9
MCS moderate	78	170	5.1	11.2	17	36	5.4	11.4
MCS loose	356	960	9.8	26.4	63	136	8.4	18.1
								
Cell cycle								
EarlyRepSeg	1,124	2,989	16.4	43.5	161	495	16.4	50.4
MidRepSeg	1,190	3,352	15.4	43.2	270	797	16.4	48.3
LateRepSeg	1,110	3,345	13.9	41.9	300	819	11.3	31.0

Both datasets (Encyclopedia of DNA Elements [ENCODE] and that reported by Bhangale and coworkers [19]) are based on a subset of 8 African Americans (the Baylor samples). bp, base pairs; CDS, coding sequence; CI, confidence interval; DHS, DNAse hypersensitive sites; ENCODE, Encyclopedia of DNA Elements; FAIRE, formaldehyde assisted isolation of regulatory elements; kb, kilobases; MCS, multi-species conserved sequence; NHGRI, National Human Genome Research Institute; transfrag, transcribed fragment; RACEfrag, rapid amplification of cDNA ends fragment; SNP, single nucleotide polymorphism; TAR, transcriptionally active region; TSS, transcription start site; TUF, transcripts of unknown function; UTR, untranslated region.

## Discussion

This work represents the first systematic description of small insertion/deletion human polymorphism data in relation to functional and evolutionary annotation, which complements larger scale structural variation data across the genome [2,21-24]. In order to understand the potential contribution made by indels to human genetic variation, we contrasted small indel rate variation by type of ENCODE region (manual or random selection), indel rates by functional annotation features, and indel rates by evolutionary constraint scores and neutral (AR) sequence; finally, we compared indel and SNP rates and their relative pattern of distribution across genomic features.

Overall, indel rates do not vary significantly between manual and randomly selected regions, suggesting that the ENCODE selection criteria for manual regions (the presence of well studied genes and availability of substantial comparative sequence) do not preclude similar genomic profiles for manual and random regions, with stratified randomly selected regions designed to be representative of a broad range of the genome [11].

Small indels are common and constitute approximately 15 insertions/deletions every 100 kb or, in terms of sequence length, 43 bp per 100 kb of the genome. The number of validated common SNPs is observed to be about seven times the number of small indels (indels per 100 kb) or twice the observed indel bp rate (bp per 100 kb). Indel rates are greatly reduced in regions associated with known functionality (largely coding DNA) and under evolutionary constraint. Compared with the overall mean, indel event rates are reduced by factors of about 20 for exon coding regions, about 5 for strict MCS sequence, and about 2 for measures of chromatin mediated regulatory sites. These observations are consistent with estimates from other studies [1,2,8]. The corresponding reduction in indel rates for these data compared with bulk DNA and when measured as indel bp per 100 kb rather than indel events, about 60 (CDS), about 10 (strict MCS), and about 2 (FAIRE and DHS).

Approximately 5% of the ENCODE sequence is estimated to be subject to moderate evolutionary constraint across mammalian species (Table 2), but only a minority of these constrained sequences are estimated to overlap with known protein coding exons and their associated UTRs (about 40%). The majority either overlap with known noncoding functional features (20%) or are suspected to be associated with previously unrecognized (40%) noncoding transcription [25].

As expected, coding (CDS, TSS, and RACEfrags) and constrained sequence (MCS) show the most constrained indel rates, followed by noncoding transcripts (transcriptionally active regions/transcribed fragments) and regulatory features (FAIRE sites, DHS, and HisPolTaf). To the extent that indels arise in functional sequence, in general indels appear to be subject to purifying selection, with indel rates negatively correlated with past evolutionary constraint across mammal and primate sequences (MCS human-mammal and GERP human-primate scores; Figures 1 and 2).

An apparent exception to the negative relationship between indel rates and constraint score is the HOXA cluster (ENCODE region 10), which runs counter to this trend. This region simultaneously exhibits the highest evolutionary constraint in the comparison of mammalian sequence (MCS) and the third highest indel rate for all the ENCODE regions (Figure 1). However, the HOXA cluster is in the centre of the region and is surrounded by gene deserts with limited evidence of evolutionary constraint. Hence, the explanation for this potentially counterintuitive observation is probably that the indel polymorphisms are largely confined to the gene deserts, whereas the constrained sequence is confined to the central portion of the HOXA cluster.

AR sequence rates are negatively correlated with mammalian sequence constraint (MCS; Figure 5), which is expected because AR sequence is neutral and not subject to natural selection. However, AR is not associated with GERP human-primate and GERP human-mammal scores (data not shown), because AR sequence was defined and identified in relation to broad mammalian sequence comparisons and not specifically primate sequence.

Multi-species constrained scores for mammals (MCS modest) and GERP for human-primate comparisons are strongly negatively correlated (Figure 6). The nonlinear relationship also reflects the fact that relatively recent (human-primate) sequence constraint comparisons fail to discriminate between the shared, more highly conserved sequences, which are only observed using broader phylogenetic comparisons.

Indel and SNP rates do not vary by the timing of DNA sequence replication during S-phase (the synthesis of DNA in preparation for mitosis) when classified as early, mid, and late S-phase replication timing [19].

Based upon two assumptions, we anticipated AR sequence rates to be positively correlated with indel rates across the ENCODE regions. What we in fact observe is a negative correlation between AR and indel rates (Figure 3). This unexpected result initially suggests that one or both of the assumptions may be false. The first assumption is that AR sequence is effectively functionless (and therefore neutral sequence), and the second is that indel mutations arise at the same rate in AR sequence as elsewhere in the genome. Although there is evidence that interspersed repeats in mammalian genomes may acquire functional roles as both protein-coding and transcriptional regulatory regions, only about 5% of the total amount of nonexonic constrained sequence (GERP) in the ENCODE regions is estimated to overlap with AR sequence [26]. This indicates that most AR sequence is still likely to be neutral and, for the most part, is unlikely to be subject to selection. By contrast, a lack of uniform indel mutation rates across the genome is more plausible [8]. Just as nucleotide point mutation rates [27] and segmental duplications [28] vary widely across the genome, it has been shown that the rate (and perhaps mechanism) of indel generation also varies widely across the genome [8].

Alternatively, the observed reduction in AR indel rates could in part arise from confounding caused by local GC content or experimental ascertainment bias. We observed AR rates to be negatively correlated with GC content (Figure 6; *r *= -0.55, *P *= 0.001) and ENCODE indel rates to be positively correlated with GC content (Figure 7). The overall indel event rate when adjusted for mean centered GC content remains unaltered, at 15 events per 100 kb (99% CI 13.4 to 16.7), whereas AR indel rates are about 4.7 events (99% CI 3.5 to 6.4) and about 10.4 events (99% CI 8.6 - 12.4) for sequence with GC content above 50% and GC content below 50%, respectively. However, although indel rates are associated with local GC content, the latter only partly accounts for reduced AR indel rates, because the indel rate for AR with reduced GC content (10.4 per 100 kb for sequence with <50% GC content) is still lower than indel rates for bulk DNA (15 per 100 kb).

One final possibility for the observed indel rate reduction for these data in AR regions, could also be an artefact of the data generation process. Ascertainment bias could arise against AR sequence with common indels because of the nature of identifying ancestral repeats common to mammalian species. Indels arising in AR sequence would reduce the alignment score used to identify ancestral repeats, so that true AR containing indels would be less likely to be identified as AR. The problem could be exacerbated if indel rates are elevated in regions that have also experienced increased rates of indel changes throughout mammalian evolution (which is likely to be the case because lineage-specific rates of indel divergence between mammals are strongly correlated with genomic region). Both of these mechanisms would give rise to experimental artefact and apparent reduction in AR indel rates.

Some of the annotation features that show significantly reduced indel rates in this analysis also show reduced levels of nucleotide substitutions (for example, CDS, TSS, RACEfrags, and MCS), indicating that selective constraint is acting to both reduce SNP as well as indel density. However, other categories such as noncoding transcription sites (transcriptionally active regions/transcribed fragments, pseudo-exons) and chromatin regulatory elements, as assessed by DHS (activated *cis*-regulatory elements in mammalian genomes) and FAIRE sites, appear to show reduced indel rates, but not reduced SNP density. This observation is consistent with experimental data that show DNA regulation of nucleosome stability to be diffuse, cumulative across base pairs, and apparently on the scale of a single nucleosome, at about 200 bp [14]. In this context, indels may have important implications for understanding genome function and variation, because chromatin composition plays a central role in regulating all DNA templated processes, including transcription, recombination, repair, and replication.

There are two potential limitations of the present study. The first relates to the completeness and accuracy of the indel and genomic annotation data [19], ensuring which is a continuing exercise for coding and noncoding transcript features [29]. Although the complete accuracy of annotations is essential to the future success of genomic and complex trait research [30], in this study we have deliberately taken a conservative statistical approach to investigating the distribution of indels in relation to annotation features, in order to account for inherent uncertainty, both in terms of biology and experimental measurement error. We also used independent data from Bhangale and coworkers [20] to compare indel rates by functional annotation and evolutionary constraint. Table 5 shows similar overall rates and reduction in indel rates for coding sequence (CDS, TSS, and RACEfrags), but not for pseudo-exons or UTRs. The data from Bhangale and coworkers also show reduced rates for open chromatin features (FAIRE and hypersensitive sites).

The second potential limitation is that, for most of our analyses, we have used summary measures for each ENCODE region, and it is likely that some effects of interest in small sequences will therefore be overlooked. Nevertheless, relatively crude summary measures by region and annotation feature still reveal clear trends between indel rates and indirect (experimental and computational) measures of functional and evolutionary constraint. We assessed the robustness of our results to various potential biases by conducting several sensitivity analyses. For instance, some of the encode regions (ENm010, ENm013, ENm014, ENr112, ENr113, ENr123, ENr131, ENr213, ENr232, and ENr321) were genotyped more intensively than others, but we found no evidence that these regions yielded substantially different results in our analyses. Indels are predominantly (58%) 1 bp in length, and we repeated analyses with only those indels with lengths in excess of 1 bp, and found that the trends in our analysis do not substantially alter (data not shown). We also repeated the analyses for insertions and deletions separately and reached the same conclusions.

## Conclusion

Small indels that arise in functional sequence are likely to be subject to negative selection, as shown by the reduced indel rates in transcribed DNA, evolutionarily constrained sequence, and - to a lesser extent - regulatory elements. Although reduced indel and SNP rates are both clearly related to coding sequence constraints, constrained indel rates in regulatory regions may reflect that indels are more likely than SNPs to moderate the structural function of regulatory elements. Indels may play a more important role than SNPs in contributing to natural genetic variation at regulatory sites, and hence they could be an important source of variation in gene expression levels.

## Materials and methods

The ENCODE project aims to identify and catalog all functional elements, including coding sequences of genes and noncoding DNA, in the human genome. A pilot study phase considered 44 discrete regions that encompass 30 megabases, or about 1% of the human genome, with 14 of these regions (about 15 megabases) selected manually and the remainder randomly [11]. Small indels in the ENCODE regions were called from shotgun re-sequencing reads and traces of the SNP discovery efforts from both the SNP consortium and the HapMap (see the report from the ENCODE Project Consortium [19] for details of discovery and validation procedures). The shotgun technology used identified indels with a maximum length of 20 bp. Whole genome sequence data were generated totalling onefold coverage of the human genome from DNA derived from a pool of cell lines from eight unrelated adult African Americans (four male and four female) enrolled in Houston, Texas, USA [31]. The SSAHADIP software package, a modification of SSAHASNP [32], was used to align these reads to build 35 of the human reference sequence, generating polymorphism calls, while keeping track of the total bases aligned for each read. In brief, the neighbourhood quality standard base alignment method was adapted to identify indels by requiring the inserted/deleted bases and the flanking five bases on either side of the indel to exceed a minimum Phred quality score of 22. If these minima were not met, then the indel was not reported.

For this study, indels and SNPs were called using the eight Baylor samples in order to facilitate comparison. Only validated SNPs (those with heterozygosity scores) were used.

As part of the HapMap project [33], ten ENCODE regions had in-depth SNP discovery by polymerase chain reaction re-sequencing on 48 individuals in four populations; this dataset represents the deepest multi-megabase resequencing data currently available and is about three times as dense as phases I and II of the HapMap project. Experimental data, sequence conservation, and feature definitions were obtained from the three experimental groups of the ENCODE Consortium and the multiple sequence analysis group [19]. All data used in our work are available at the ENCODE project at University of California, Santa Cruz [34] and are available from the corresponding author. The full set of ENCODE indels can be downloaded directly [35].

To evaluate the potential contributions of insertion and deletion events to functional variation, we calculated indel density as a percentage of nucleotides for each ENCODE region and classification feature. Genomic coordinates for features and ENCODE regions were used to estimate two summary measures of indel density: the number of indels per 100 kb of the region length or total feature, and the number of indel bp per 100 kb for the region length or total feature. The densities were analyzed using a negative binomial model with the number of indels or base pairs as the response, the lengths of sequence as an offset, and data aggregated to the region level [36]. This approach allowed us to calculate 99% confidence intervals for indel and SNP densities, compensated for potential over-dispersion, and provided a conservative framework for testing for differences between manually and randomly selected regions and genomic features. Comparisons across genomic features are also likely to be conservative, because confidence intervals were generated using aggregate summary measures across ENCODE regions, rather than raw data. The SNP densities correspond to a measure of heterozygosity (1 SNP per 100 kb, corresponds to a heterozygosity of 1 × 10^-5^).

Comparative sequence analysis has become a key bioinformatics tool for identifying noncoding functional DNA [37]. We use two derived scores that attempt to measure the relative evolutionary constraint of DNA sequences: the lengths of MCSs determined from the multiple sequence alignments comparing human with 13 species of mammal [25], and rejected substitution or GERP scores from comparisons between humans and primates [26].

Evolutionarily constrained sequences were identified using three independent sequence conservation constraint programs (binCons, phastCons, and GERP) for three different multiple sequence alignments generated using TBA, MLAGAN, and MAVID for 14 mammalian species [25]. Each alignment used human sequence as the reference. Three levels of MCS were defined: strict, in which sequences are constrained in all alignment/conservation combinations; moderate, in which sequences are constrained in at least two of three alignments, and from two of three conservation programs; and loose, in which sequences are constrained in at least one alignment/conservation combination. We found results did not alter qualitatively between use of the three scores and we present results using moderate MCS in this report. Note that we refer to 'constrained' rather than 'conserved' sequence because conservation *per se *does not imply function, whereas constraint does. GERP identifies regions at high resolution that exhibit nucleotide substitution deficits, and measures these deficits as 'rejected substitutions'. Rejected substitutions reflect the intensity of past purifying selection and are used to rank and characterize constrained elements. GERP scores are positive in constrained regions and negative in neutral DNA [26], and MCS scores are high in constrained regions and low in neutral DNA [25].

To illustrate potential relationships between indel rates and constraint scores, summary data for the 44 ENCODE regions were plotted using cubic smoothing splines and robust linear regression using an M estimator [36]. The latter approach is robust to potential outliers but conservative. Potential outliers were also identified using standard regression leverage-residual diagnostics [36], and we assessed the sensitivity of results to outlier removal using Pearson product moment correlation coefficients (*r*) and adjusted linear *R*^2 ^statistics (multiple correlation coefficient *R*).

## Abbreviations

AR, ancestral repeat; bp, base pairs; CDS, coding sequence; CI, confidence interval; DHS, DNAse hypersensitive sites; ENCODE, Encyclopedia of DNA Elements; FAIRE, Formaldehyde assisted isolation of regulatory elements; GERP, genomic evolutionary rate profiling; HOXA, HOXA cluster (homeobox gene family); kb, kilobases; MCS, multi-species constrained sequence; RACEfrag, rapid amplification of cDNA ends fragment; SNP, single nucleotide polymorphism; TSS, transcription start site; UTR, untranslated region.

## Authors' contributions

TC and TA analysed the indel data and wrote the manuscript. GC generated the evolutionary constraint scores and JM generated the shotgun indel data. EM, GC, JM and DB provided detailed advice and commented on the text. All authors read and approved the final version of the manuscript.
